# Empirical evaluation of variant calling accuracy using ultra-deep whole-genome sequencing data

**DOI:** 10.1038/s41598-018-38346-0

**Published:** 2019-02-11

**Authors:** Toshihiro Kishikawa, Yukihide Momozawa, Takeshi Ozeki, Taisei Mushiroda, Hidenori Inohara, Yoichiro Kamatani, Michiaki Kubo, Yukinori Okada

**Affiliations:** 1 Department of Statistical Genetics, Osaka University Graduate School of Medicine, Suita, 565-0871 Japan; 2 Department of Otorhinolaryngology - Head and Neck Surgery, Osaka University Graduate School of Medicine, Osaka, 565-0871 Japan; 3 Laboratory for Genotyping Development, RIKEN Center for Integrative Medical Sciences, Yokohama, 230-0045 Japan; 4 Laboratory for Pharmacogenomics, RIKEN Center for Integrative Medical Sciences, Yokohama, 230-0045 Japan; 5 Laboratory for Statistical Analysis, RIKEN Center for Integrative Medical Sciences, Yokohama, 230-0045 Japan; 6 Kyoto-McGill International Collaborative School in Genomic Medicine, Kyoto University Graduate School of Medicine, Sakyo-ku, Kyoto, 606-8507 Japan; 7 RIKEN Center for Integrative Medical Sciences, Yokohama, 230-0045 Japan; 8 Laboratory of Statistical Immunology, Immunology Frontier Research Center (WPI-IFReC), Osaka University, Suita, 565-0871 Japan

## Abstract

In the design of whole-genome sequencing (WGS) studies, sequencing depth is a crucial parameter to define variant calling accuracy and study cost, with no standard recommendations having been established. We empirically evaluated the variant calling accuracy of the WGS pipeline using ultra-deep WGS data (approximately 410×). We randomly sampled sequence reads and constructed a series of simulation WGS datasets with a variety of gradual depths (n = 54; from 0.05× to 410×). Next, we evaluated the genotype concordances of the WGS data with those in the SNP microarray data or the WGS data using all the sequence reads. In addition, we assessed the accuracy of HLA allele genotyping using the WGS data with multiple software tools (PHLAT, HLA-VBseq, HLA-HD, and SNP2HLA). The WGS data with higher depths showed higher concordance rates, and >13.7× depth achieved as high as >99% of concordance. Comparisons with the WGS data using all the sequence reads showed that SNVs achieved >95% of concordance at 17.6× depth, whereas indels showed only 60% concordance. For the accuracy of HLA allele genotyping using the WGS data, 13.7× depth showed sufficient accuracy while performance heterogeneity among the software tools was observed (the highest concordance of 96.9% was observed with HLA-HD). Improvement in HLA genotyping accuracy by further increasing the depths was limited. These results suggest a medium degree of the WGS depth setting (approximately 15×) to achieve both accurate SNV calling and cost-effectiveness, whereas relatively higher depths are required for accurate indel calling.

## Introduction

The innovation of next-generation sequencing (NGS) technologies has brought lower costs, higher throughput, and expansion in scales of human genome sequencing. The target regions of human genome sequencing have expanded from specific regions or whole exome into whole genome. Whole-genome sequencing (WGS) was developed as an approach capable of assessing all the nucleotide sequences of an individual’s genome^1,2^. Thus far, it has contributed to the identification of many disease-related mutations. Recently, the use of WGS has become widespread in medical facilities as well as research laboratories^3^. The clinical application of WGS is gaining importance toward the achievement of personalized medicine^3,4^.

In the study design of WGS, the setting of a sequencing depth is a crucial parameter in defining the variant calling accuracy and the study cost. Several studies have recommended the depth setting to optimize the balance between accuracy and cost. When NGS was initially applied for human genomes, relatively high read depths were recommended (approximately 33×–35× for SNVs and 60× for indels)^5–7^ by assessing the numbers of the called variants and the concordance rates with reference data such as SNP microarrays. Such deep sequencing approaches successfully identified causal variants in the studies of rare Mendelian disorders^8,9^. In addition, deep WGS permitted new insights into the diversity of the human genome, its structure, and the genetic history of the populations worldwide^10–13^.

On the other hand, the analysis of common diseases requires relatively larger sample sizes to obtain sufficient statistical power to detect risk variants. Thus, the implementation of deep WGS for all the subjects enrolled in common disease studies is challenging because of the high sequencing costs and the requirement for abundant machine resources. The 1000 Genomes Project^14^ reported that even with a low depth, high accuracy could be achieved by utilizing multi-sample joint calling and genotype imputation. This project adopted the study design of sequencing a large proportion of samples at a relatively low depth (average, 3.7×). Recently, the UK10K project^15^ performed WGS of 3,781 subjects at 7× depth. Furthermore, Pasaniuc *et al*. reported that a combination of extremely low-depth WGS (0.1×–0.5×) followed by whole-genome genotype imputation could reveal a sufficient amount of variants required for the implementation of GWAS^16^.

Currently, discussions regarding an appropriate depth setting in WGS still remain controversial. To date, WGS studies have been reported at various depth settings, from low depths of approximately 1.0×–5.0× to depths as high as 30×^10,17^. In recent years, detailed evaluation of WGS accuracy has been conducted using gold standard datasets such as NIST Genome in a Bottle and Platinum Genomes^18,19^. These studies generated high-confidence, genome-wide variant sets, but the transition in accuracy according to the gradual variation of sequencing depths for identifying the appropriate depth has not been evaluated. Advances in sequencing technologies such as library preparation methods and WGS data analysis pipelines are additional areas where the refinement of standard WGS depth setting for current genome studies is required. Furthermore, highly accurate variant calling of an individual’s genome sequences is required for the utilization of their genome information in clinical and diagnostic applications, such as personalized medicine^20^. Notably, several genetic loci with strong clinical manifestations have complex and diverse genome structures (e.g., human leukocyte antigen [HLA] genes in the major histocompatibility [MHC] region), which makes accurate WGS variant calling particularly challenging^21^.

In this study, we empirically evaluated an individual’s variant calling accuracy of the WGS pipeline by utilizing ultra-deep WGS data (approximately 400×). By randomly sampling the sequence reads from the ultra-deep WGS data, we constructed a series of simulation WGS datasets with a gradual variety of depths (from 0.05× to 400×). WGS data of each depth were evaluated by calculating the genotype concordances of the called variants and those obtained from the SNP microarray or those of the WGS data with all the reads and by the accuracy of HLA genotyping.

## Results

### Summary of the WGS data sets

**Figure 1:**
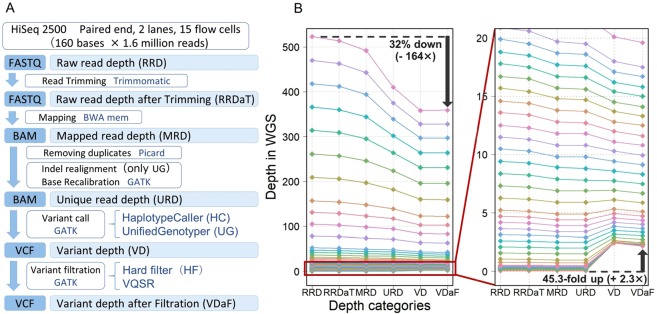
Process of whole-genome sequence variant calling. (**A**) Summary of the WGS variant calling pipeline. The pipeline was based on GATK (version 3.6) Best Practices. We adopted the two major variant callers and two variant filtration procedures. (**B**) Transition of the depths along with the WGS variant calling process. We assessed a total of six depth stages (RRD, RRDaT, MRD, URD, VD, and VDaF).

We selected one Japanese sample from the BioBank Japan Project^22,23^. We conducted an ultra-deep WGS of the subjects using the Illumina HiSeq. 2500 platform (see details in Methods). Sequence reads were aligned to the reference human genome with the decoy sequence (GRCh37/hg19, hs37d5). By randomly sampling the mapped reads, we constructed a series of simulation WGS datasets with a wide range of gradual depths in 54 levels ranging from 0.05× to 410×. The WGS variant calling analysis pipeline was primarily based on GATK (version 3.6) Best Practices^24^ (Fig. 1A). For the comparisons, we adopted the two major variant calling pipelines (UG and HaplotypeCaller [HC]) as well as the two variant filtration procedures (VQSR and HF).

In the process of the WGS variant calling, we assessed a total of six depth stages along with the conversion from the raw sequence data (FASTQ file format) to the final variant calling (VCF file format; Fig. 1A). In general, the WGS data demonstrated a reduction of the depths along with the variant calling process, with higher depths demonstrating higher reduction rates (e.g., 31.4% reduction from 523× of the FASTQ format to 359× of the VCF format vs. 13.3% reduction from 10.5× to 9.1×; Fig. 1B). The reduction was most apparent at the duplicate read removal process (MRD to URD). Notably, the low-depth WGS data (approximately <5.0×) showed an increase in the depths in the last two stages of the variant calling pipeline because of exclusion of the variant sites with low quality, in which the depths were likely to be considerably lowered (e.g., 45.3-fold increment of the depth from 0.052× of URD to 2.36× of variant depth after filtration [VDaF]).

### WGS variant calling results based on depths

**Figure 2:**
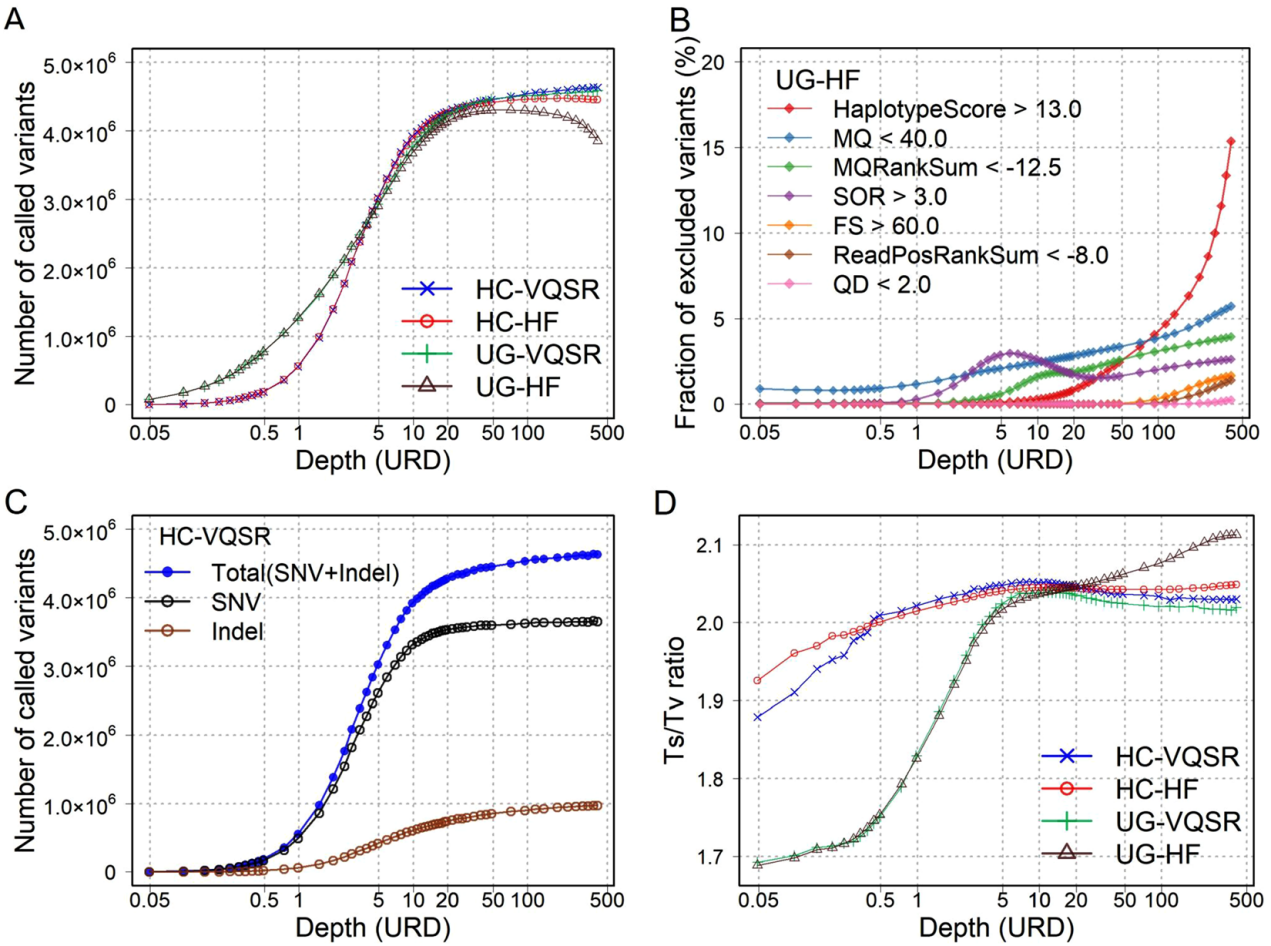
Variant calling results of the WGS data simulation according to the depths. (**A**) Number of called variants based on depths. The x-axis shows the depth of each WGS data in a logarithmic scale. Each line shows the data of the combination of variant callers and variant filtrations: HC, HaplotypeCaller; UG, UnifiedGenotyper; VQSR, Variant Quality Score Recalibration; HF, hard filter. (**B**) Exclusion rates for each filtration item of HF. The data were called by UG and filtered by HF. (**C**) Number of called SNPs and indels based on depths. The data were called by HC and filtered by VQSR. (**D**) Ts / Tv ratio based on depths.

For the empirical evaluation of the WGS variant calling accuracy, we adopted the BAM file depth after duplicate read removal (i.e., URD) as a standard depth metric. Expectedly, the number of called variants increased according to depths and became almost flat (approximately 4.3 × 10^6^) at depths of approximately ≥20× (Fig. 2A). We found that differences in the number of variants between variant callers (HC and UG) were more apparent than those between variant filtrations (VQSR and HF). In general, UG called a higher numbers of variants than HC at low depths (<5×). We note that the combination of UG and HF demonstrated a remarkable decrease in the number of called variants at high depths (>100×). Accordingly, we assessed an exclusion rate for each filtration item in the variant calling process of UG and HF (Fig. 2B). We found that the exclusion rates for the “HaplotypeScore” item increased particularly at high depths (>100×), attaining values above 15% at 410× depth (Fig. 2B). HaplotypeScore quantifies the estimated haplotype numbers of each region, which are originally defined as two (i.e., diploid). Increases in the HaplotypeScore may reflect overestimation of haplotypes resulting from an increase of the mapped reads with marginally different sequences due to error. Because the filtration could be excessive in the combination of UG and HF, the CRs of the called variants with the SNP microarray data decreased at high depths (Fig. S1A).

We then assessed the numbers of the called variants for SNVs and indels separately. The number of SNVs became flat at approximately 20× depth, whereas the number of indels showed gradual increases even at over 100× depth (Fig. 2C). As for the transition / transversion (Ts / Tv) ratio, the difference between variant callers was more apparent than variant filtrations, too (Fig. 2D). While UG showed low Ts / Tv ratios (<2.0) at low depths (<5×), the Ts / Tv ratio in HC was relatively high even at low depths (>2.0 at 0.5×).

### Genotype concordance comparison with SNP microarray

**Figure 3:**
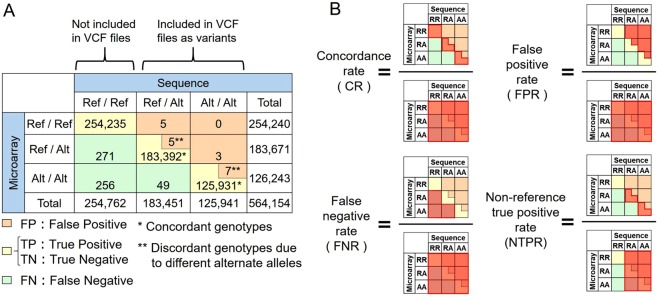
Definition of the genotype concordance measurement. (**A**) Genotype concordance matrix. All combinations of the three SNV genotypes (reference / reference [Ref/Ref], reference / alterative [Ref/Alt], alternative / alternative [Alt/Alt]) between the WGS and SNP microarray were assigned. The positions were classified as false positive when alternate alleles were discordant between the sequence and the microarray data. We assessed genotypes as homo-reference that were not included in the VCF files of the WGS data. (**B**) Genotype concordance metrics. We adopted all the genotypes on the microarray as a parameter during assessment of CR, FPR, and FNR, whereas variants called in each of the WGS data were used to assess NTPR.

We obtained genome-wide SNP genotypes of the same individual by using the high density SNP microarray (Illumina HumanOmniExpressExome-8 v1.2)^25,26^. We checked the variant quality (see details in Methods) and selected 564,154 SNPs for evaluation. Using the SNP microarray data as “correct answers”, we empirically evaluated the accuracy of the SNV calling in the simulation WGS datasets. For each simulation WGS data, we calculated a 3 × 3 contingency table in which all combinations of the three SNV genotypes between the WGS and SNP microarray were assigned (Fig. 3A). Subsequently, we estimated four indices to evaluate the genotype concordances: CR, FPR, FNR, and NTPR; Fig. 3B).

**Figure 4:**
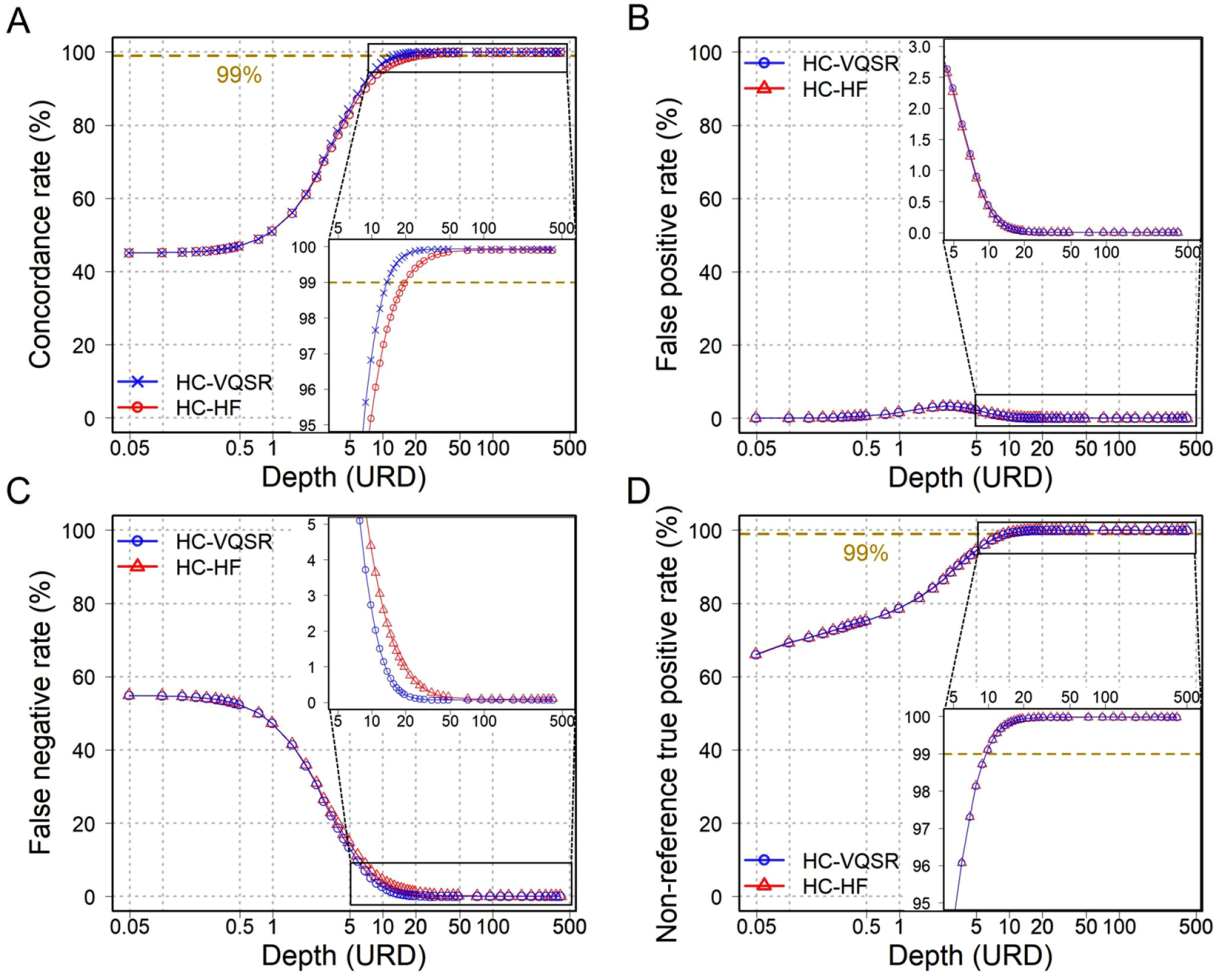
Comparison of genotype concordances with SNP microarray. The x-axis shows the depth of each WGS data in a logarithmic scale. The y-axis corresponds to each metric of Fig. 3B. (**A**) CR based on depths. With the settings of HC and VQSR (blue line), >13.7× depth achieved as high as >99% of concordances (ocher broken line), whereas HC and HF (the red line), >21.9× depth achieved. (**B**) FPR based on depths. (**C**) FNR based on depths. (**D**) NTPR based on depths. With both filtration procedures of HF and VQSR, >9.8× depth achieved as high as >99% of NTPR (ocher line).

Expectedly, the WGS data with higher depths showed higher CRs. Empirically, >13.7× depth achieved as high as >99% of concordances (with the settings of HC and VQSR; Fig. 4A–C and Table 1). As for the SNP with heterozygous genotypes in the SNP microarray, >18.6× depth achieved as high as >99% concordance (Fig. S2 and Table S1). On comparison of the CRs between variant filtrations, VQSR showed marginally higher CRs and lower FNRs than HF at 3×–50× depths (Fig. 4A). As for variant callers, HC showed higher FNRs and lower FPRs than UG data at low depths (Fig. S1A–C). As described above, the combination of UG and HF showed an increase in the FNRs and a decrease in the CRs at high depths (Fig. S1A). To evaluate the true positive rates, we then confined the genomic sites where variants were called in each of the WGS data, and observed that >9.8× depth achieved as high as >99% of NTPRs (Fig. 4D). These results empirically suggest that WGS data with a medium degree of depth setting (approximately 15×) could achieve SNV calling with high accuracy. We classified SNPs into five levels (≥0.5, 0.1–0.5, 0.05–0.1, 0.01–0.05, and <0.01) of alternate allele frequencies (AAF). We obtained allele frequencies data of WGS of the Japanese population from BBJ (n = 1,026; http://jenger.riken.jp/data)^13^. SNPs of low AAF showed high concordance rates at low depths because the true negative alleles were more than false negative alleles at low AAF (Fig. S3). On the other hand, SNPs of High AAF showed high Non-reference true positive rates at low depths because homo-alternate alleles were more than heterozygous at high AAF. At 14.7× depth, the differences between each AAF data were slight (Table S2).

**Table 1:** Summary of WGS data’s comparison.

		HaplotypeCaller		UnifiedGenotyper	
		VQSR	Hard Filter	VQSR	Hard Filter
Comparison with SNP microarray data	Concordance rate > 99%	13.7×	21.9×	13.7×	21.9×
	True positive rate > 99%	9.8×	9.8×	9.8×	9.8×
Comparison with WGS all reads data	SNV concordance rate > 95%	17.6×	16.6×	34.0×	13.7v
	Indel concordance rate > 95%	339×	339×	264×	264×

### Comparison of genotype concordance with the WGS data using all the sequence reads

**Figure 5:**
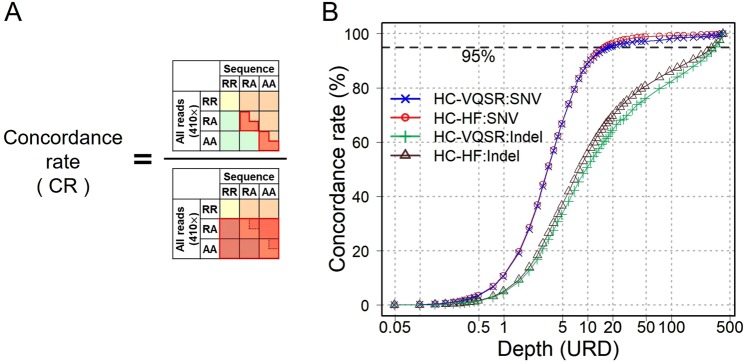
Comparison of genotype concordance with WGS data of all the reads. (**A**) Definition of concordance rates in comparison with the WGS data of all the reads. We adopted variants called in the WGS data of all the reads as a parameter. (**B**) Concordance rates based on depths. With the HC and VQSR settings (blue line), SNVs of >17.6× depth achieved as high as >95% of concordances (broken line), whereas HC and HF (the red line) >16.6× depth. On the other hand, indels required >339× depth.

Because the SNPs on the SNP microarray are empirically known to be sequenced with relatively higher accuracy^13^, we then assessed CR of whole-genome SNVs and indels separately. To this end, we adopted the ultra-deep WGS dataset using all the sequence reads (410×) as “correct answers” and empirically evaluated the CR, FPR, FNR, and NTPR of each of the simulation WGS datasets as indicated in Figs 5A and S4. Each dataset was in the process of the same variant calling and the same variant filtration as the 410× dataset. Considering that the WGS variant calling using all the reads may still have uncertainty, such as stochastic processes in the variant calling pipeline (e.g., VQSR), we adopted the less stringent threshold of 95% of CR for assessment.

As a result, SNVs achieved >95% of CR with 17.6× depth (with HF and VQSR; Fig. 5B). Conversely, indels showed only 60% of CR at this depth, and much higher depths (>339×) were required to achieve 95% concordances. These results emphasize that the WGS data with a medium degree of depth setting (approximately 15×) can be considered as one of the recommendation for SNV calling, whereas much higher depths are required to achieve accurate indel calling.

### Accuracy of HLA allele genotyping using WGS data

Variations of HLA genes have strong genetic risks on a wide range of human complex traits^27^, and accurate genotyping of HLA alleles using WGS data is considered an essential step for the implementation of personalized medicine. However, because the genetic architecture of the MHC region is complex and highly polymorphic, its variant calling is relatively challenging when compared to that of other genetic loci^28^. To date, a variety of software tools have been developed to genotype HLA alleles using WGS data; however, the empirical evaluation of their performances in terms of genotyping accuracy requires further assessments. Recently, the in silico imputation of HLA alleles from the surrounding SNP genotype data has emerged as a cost-effective approach to obtain information on HLA alleles^29^.

In this study, we adopted four major software tools that genotype HLA alleles using sequence reads obtained from WGS: PHLAT^30^, HLA-VBseq.^31^, HLA-HD^32^, and HISAT-genotype^33^ and estimated 4-digit classical alleles of the class I and II HLA genes (n = 8) for each of the simulation WGS datasets. We also adopted an HLA imputation software tool of SNP2HLA^29^ and an imputation reference panel of the Japanese population^34,35^. We note that the concept of HLA imputation is different from that of HLA calling. To identify the HLA genotype, HLA imputation software utilizes the SNV genotypes of the WGS datasets for imputation of the HLA alleles, while HLA calling software directly utilizes the sequencing reads of the WGS datasets for each software’s original alignment. As “correct answers,” we obtained 4-digit alleles of the HLA genes using the SSO or SBT methods and assessed the concordance.

**Figure 6:**
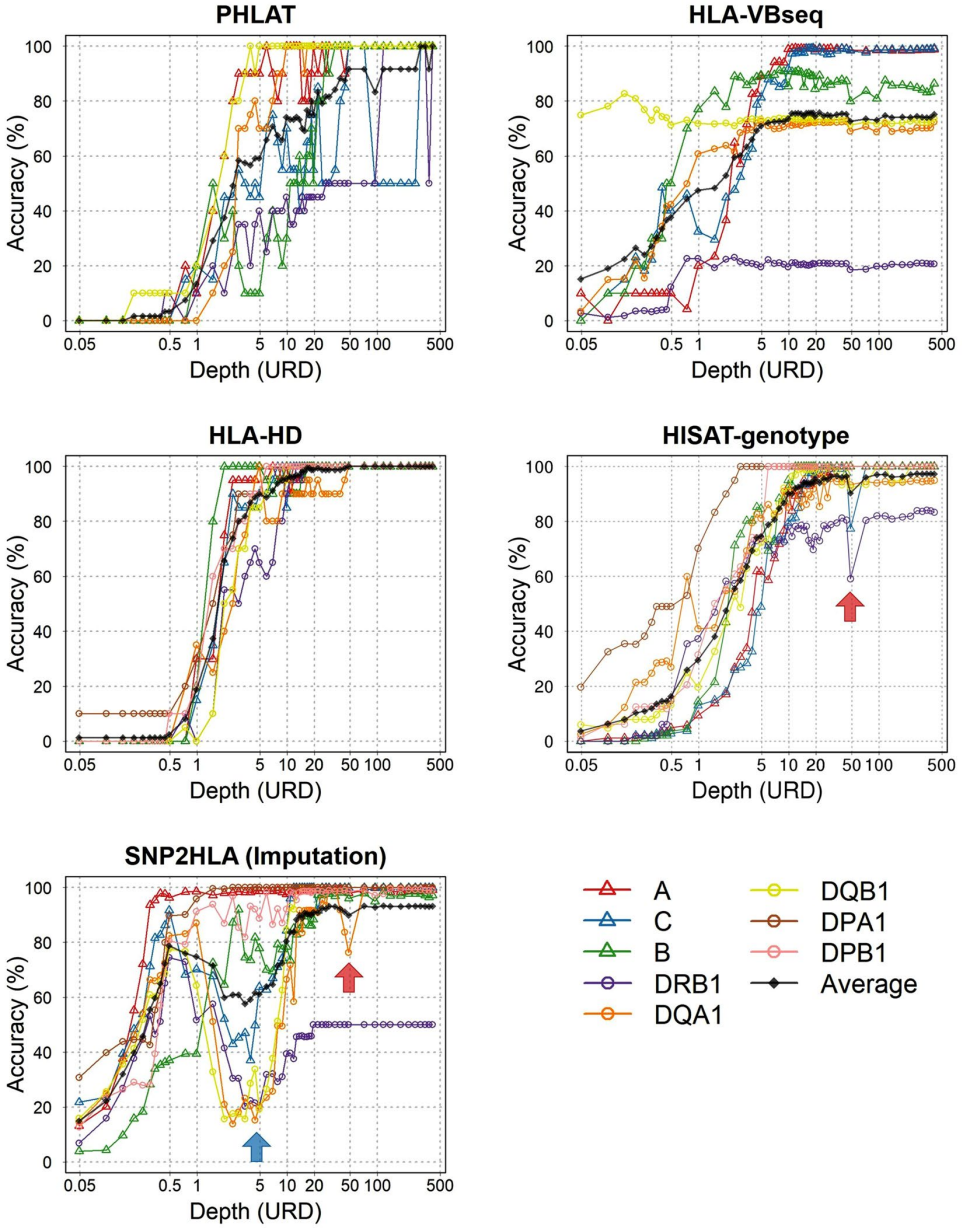
Accuracy of HLA genotyping using WGS data. The black line indicates the average accuracy of each HLA gene allele. HLA-HD, HISAT-genotype, and SNP2HLA genotyped the alleles of the all the classical HLA genes, while *HLA-DPA1* and *HLA-DPB1* were not available at PHLAT and HLA-VBseq. The red arrow indicates the decrease of the accuracy at 48× depth. This is because the accuracy under 45× depth was average of 10 datasets of different random seeds, while the accuracy over 45× depth was from one data. The blue arrow indicates the decrease of the accuracy of SNP2HLA at around five depth. This reflects high false positive rates of SNV calls at those depths (Fig. 4B), because SNP2HLA utilizes VCF files and is affected by the accuracy of SNV calls in HLA genes.

Similar to the findings of the SNV and indel variant calling, the WGS datasets with higher depths showed higher accuracy in HLA allele genotyping in the four software tools (Fig. 6 and Table 2). PHLAT and HLA-VBseq showed heterogeneity of typing accuracy among the HLA genes, and only around 75% of concordances at >13.7× depth, with the genome-wide SNV calling of WGS achieving >99% accuracy. In turn, HLA-HD and HISAT-genotype achieved a relatively more accurate performance (an average CR of 96.9% and 92.9% at 13.7× depth). As for the HLA imputation software of SNP2HLA, the genotyping accuracy of the HLA-DRB1 allele (DRB1*09:01 / DRB1*14:54 heterozygote) was particularly low (45.8% at 13.7× depth) when compared to other HLA genes (70.4% on average). This was attributed to the HLA-DRB1*14:54 allele having been wrongly genotyped in the imputation reference panel as HLA-DRB1*14:01 owing to the technical limitation of the SSO method as previously reported^36^. For all software tools, improvement of the genotyping accuracy was limited above 20× depth, and even in the ultra-deep WGS datasets with over 100× depth, only HLA-HD could achieve 100% of accuracy.

**Table 2:** Accuracy of HLA genotyping using WGS data.

HLA gene	Direct genotyping	Average accuracy of 13.7× data (%)					Average accuracy of 3-digit depth data (%)				
		PHLAT	HLA- VBseq	HLA-HD	HISAT- genotype	SNP2HLA	PHLAT	HLA- VBseq	HLA-HD	HISAT- genotype	SNP2HLA
A	24:02 / 24:02	100	99.2	95.0	90.1	98.6	100	98.6	100	100	98.9
C	01:02 / 03:04	60.0	90.4	90.0	100	100	100	85.2	100	100	100
B	40:02 / 40:02	40.0	98.0	100	89.8	84.8	72.2	98.9	100	100	97.3
DRB1	09:01 / 14:54	40.0	71.1	100	78.6	45.8	100	70.3	100	82.7	50.0
DQA1	01:04 / 03:02	100	73.4	100	85.3	91.7	100	72.5	100	94.6	100
DQB1	03:03 / 05:03	100	20.4	90.0	99.4	100	94.4	20.6	100	98.9	100
DPA1	01:03 / 01:03	—	—	100	100	100	—	—	100	100	100
DPB1	02:01 / 02:01	—	—	100	100	98.2	—	—	100	100	99.0
Average		73.3	75.4	96.9	92.9	89.9	94.4	74.3	100	97.0	93.2

## Discussion

In this study, we empirically evaluated the accuracy of the WGS variant calling using ultra-deep WGS data (410×). Although long-read sequencing has become widespread, we adopted the general short-read sequencing approach on the Illumina platform in consideration of its potential application to personalized medicine. We found that relatively higher amount of depth loss (>30%) was observed in the process of variant calling for such high-depth WGS data. As for SNV calling, the number of the called variants became flat at approximately 20× depth. In comparison with the genotypes obtained from the SNP microarray, >13.7× depth achieved as high as >99% of concordances. In comparison with the WGS data using all the sequence reads, CRs above 95% were achieved with >17.6× depth. Our results suggest the medium degree of the depth (approximately 15×) as an appropriate depth setting in WGS to achieve accurate SNV genotyping. This depth recommendation is relatively lower than those previously reported (approximately ≥30×)^5,6^, which would occur because of sequencing accuracy improvements through updates in the WGS platforms and variant calling pipeline of the WGS data^24^. The threshold setting of CR with SNP microarray data is debatable. We set 99% as the threshold following previous reports, but some cases require higher accuracy at present.

On the other hand, we found that the accurate indel calling may require higher depths than those necessary for SNVs. The number of called indels for one single individual increased even over as high as >100 depth. Indels included in a genome of one individual have been estimated in a wide range^14,37,38^. While the Illumina Platinum Genome project^19^ reported 693,623 indels as the high confidence set, another study using the classical Sanger sequencing method on a large scale reported 851,575 indels per individual^39^. In our WGS data, at least 48× depth was required for calling this amount of indels. In comparison with the WGS data using all the sequence reads, a depth above 300× was required to achieve 95% of accuracy.

One characteristic of our study is the evaluation of HLA genotyping accuracy using WGS data. While heterogeneity in performance was observed among the software tools, we found that a medium degree of WGS depth setting (approximately 15×) could achieve high CR, whereas improvement in accuracy was limited when further depths were included in the WGS design. Our study also noted that WGS-based HLA typing could complement the technical limitations observed in the classical HLA genotyping methods (e.g., inaccurate calling of HLA-DRB1*14:54 as HLA-DRB1*14:01 in the SSO method).

To date, discussions on comparisons of variant caller options in the WGS analysis pipeline, such as HC/UG and VQSR/HF options in the GATK pipeline, have been controversial. For the variant caller, our study shows that HC called the variants with high quality in terms of FPR and Ts/Tv ratio at low depths (<5×). At high depths, UG was likely to remove more variants than HC, which could reduce efficiency in detecting the variants. These findings recommend HC as the primary choice in WGS analysis. As for the variant filtration, VQSR was superior to HF in the comparison with the SNP microarray, whereas HF showed higher CR than VQSR in the comparison with the WGS data using all the sequence reads. These results suggest that the recommendations on HC or VQSR depend on the situations, with the combination of HC and VQSR currently resulting in more appropriate variant calling.

We note that this study was conducted on only one subject. Although our study design to generally evaluate the WGS variant calling pipeline would be robust to the bias specific to the individual, our study did not assess the analytic protocol that requires the enrollment of WGS data from multiple individuals (e.g., a joint calling method). Also, the evaluation of the accuracy of HLA genotyping could be desirable in multiple individuals because the accuracy depends on individual type. Further studies to assess multiple individuals would be warranted.

In conclusion, our ultra-deep WGS analysis suggested a relatively medium degree of the depths as a recommendation of the WGS study design, which should be a useful indication for the future genomic studies.

## Material and Methods

### Variant calling pipeline of the WGS data

We adopted a Japanese sample from the BioBank Japan Project, a nation-wide hospital-based cohort of the Japanese population^22,23^. A WGS library was constructed using the TruSeq Nano DNA Library Preparation Kit (Illumina) and paired-end 160-bp reads on Hiseq. 2500 with rapid output run mode (2 lanes of 15 flow cells; Fig. 1A) were generated. The sequence reads were converted to the FASTQ format using bcl2fastq (version 1.8.4) and trimmed to clip Illumina adapters using Trimmomatic (version 0.33). The reads were aligned to the reference human genome with the decoy sequence (GRCh37/hg19, hs37d5) using BWA-MEM (version 0.7.15, default parameters).

By randomly sampling the mapped reads, we constructed a series of simulation WGS datasets with gradual depths in 54 levels from 0.05× to 410× using SAMtools (version 1.3.1, parameters: view –h –b –s). We set depths of the simulation WGS data at a combination of equal intervals in both linear and logarithmic scales. Considering that the stochastic bias could potentially affect the results in the WGS datasets with lower depth settings, we constructed 10 different datasets for each of the low-depth settings (<45×) by changing random seeds, and adopted average values in the analysis results to conduct the analysis in a robust manner.

The WGS variant calling analysis pipeline was based on GATK (version 3.6) Best Practices^24^. The duplicate reads were removed using Picard (version 2.5.0, default parameters). Indel realignment using GATK was performed only in situations where UnifiedGenotyper (UG) was adopted as a variant caller. Base quality score recalibration using GATK (default reference: 1000G_phase1.indels.b37.vcf, dbsnp_138.b37.vcf, and Mills_and_1000G_gold_standard.indels.b37.vcf) was performed in all situations. For comparisons, we adopted two variant callers (UG and HaplotypeCaller [HC]). While HC was conducted with default parameters, UG was conducted with “-dkv 100000” in addition to default parameter for avoidance of down sampling. Additionally, we used two variant filtration procedures: Variant Quality Score Recalibration (VQSR) and Variant Filtration as hard filter (HF).

### Definition of the depths along with the WGS variant calling processes

In the process of the WGS variant calling, we assessed a total of six depth stages (Fig. 1): raw read depth (RRD), raw read depth after trimming (RRDaT), mapped read depth (MRD), unique read depth (URD), variant depth (VD), and variant depth after filtration (VDaF). When examining the depths of the FASTQ files (i.e., RRD and RRDaT), we calculated the sums of each file’s base numbers and divided them by the number of bases in the reference sequence (GRCh37) without unknown bases. We used DepthOfCoverage of GATK to calculate the depths of BAM files, and VCFtools (versions0.1.13) for VCF files.

### Variant Filtration in the WGS variant calling pipeline

VQSR and HF were separately performed for the WGS variant calling (both SNPs and indels), in which we followed the GATK recommendations for the parameter setting. VQSR parameters were defined as follows: HapMap 3.3, Omni 2.5 SNP BeadChip, and 1000 Genomes phase I data were used for SNPs in training sets, whereas Mills-Devine and 1000 Genomes phase I data were employed for indels. Regarding variant annotations, we adopted DP, QD, FS, SOR, MQ, MQRankSum, and ReadPosRankSum for SNPs, and DP, QD, FS, SOR, MQRankSum, and ReadPosRankSum for indels. To determine the truth sensitivity threshold of VQSR, we compared the concordance rates of the SNP microarray data and the WGS data at three threshold levels (90.0%, 99.0%, and 99.9%), and ultimately selected 99.9% because of its highest concordance rate (Fig. S5). For HF, we used the following criteria: QD < 2.0, FS > 60.0, MQ < 40.0, MQRankSum < −12.5, ReadPosRankSum < −8.0, HaplotypeScore > 13.0, and SOR > 3.0 for SNPs, and QD < 2.0, FS > 200.0, ReadPosRankSum < −20.0, and SOR > 10.0 for indels.

### Concordance comparisons with SNP microarray data or the WGS data with all the reads

We obtained genome-wide SNP genotypes of the same individual by using SNP microarray (Illumina HumanOmniExpressExome-8 v1.2) as described previously^25,26^. We excluded the following variants: call rate < 0.99, HWE < 1.0 × 10^−6^, MAF < 0.01, and multi-allelic variants in 1000 Genomes Project phase 3. Using the SNP microarray data as “correct answers,” we empirically evaluated the accuracy of the SNV calling in the simulation WGS datasets. For each simulation WGS data, we calculated a 3 × 3 contingency table in which all combinations of the three SNV genotypes of the WGS and the SNP microarray were assigned (reference / reference [Ref/Ref], reference / alterative [Ref/Alt], alternative / alternative [Alt/Alt]; Fig. 3A). Next, we estimated four indices to evaluate genotype concordances: concordance rate (CR), false positive rate (FPR), false negative rate (FNR), and non-reference true positive rate (NTPR; Fig. 3B). We adopted all the genotypes on the microarray as a parameter during assessment of CR, FPR, and FNR, whereas we confined the genomic sites where variants were called in each of the WGS data during assessment of NTPR. We classified the positions into false positive when alternate alleles were discordant between the sequence and microarray data.

Regarding concordance with the WGS data with all the reads, we assessed concordance rates (CR) between the ultra-deep WGS data using all the sequence reads (410×) and the simulation WGS data of each depth. We adopted the former as “correct answers,” and evaluated concordances separately for SNVs and indels. As for SNVs, we conformed the definition of “concordance” of Fig. 3. On the other hand, we defined as “concordance” of indels when both the positions and the base sequences were concordant.

### HLA allele genotyping from the WGS data

We adopted four major software tools that genotype HLA alleles by using sequence reads obtained from WGS: PHLAT (version 1.0)^30^, HLA-VBseq.^31^, HLA-HD (version 1.0.0)^32^, and HISAT-genotype^33^ and estimated 4-digit classical alleles of the three class I (*HLA-A*, *HLA-B*, and *HLA-C*) and five class II HLA genes (*HLA-DRB1*, *HLA-DQA1*, HLA-DQB1, *HLA-DPA1*, and *HLA-DPB1*) for each of the simulation WGS datasets. We also adopted an HLA imputation software tool of SNP2HLA^29^ and the population-specific imputation reference panel of Japanese^34,35^ and imputed the HLA alleles based on the SNV genotypes of the MHC region called separately for the WGS datasets. While HLA-HD, HISAT-genotype, and SNP2HLA genotyped the alleles of all classical HLA genes, genotyping of the *HLA-DPA1* and *HLA-DPB1* alleles was not available at PHLAT and HLA-VBseq. As “correct answers”, we obtained 4-digit alleles of the HLA genes using the sequence-specific oligonucleotide hybridization (SSO) or sequencing-based typing (SBT) methods and assessed the concordance.

## Supplementary information

Supplementary Figure S1–5, Supplementary Table S1,2
