# Supplementary material for: Empirical evaluation of variant calling accuracy using ultra-deep whole-genome sequencing data
Source: Sci Rep. 2019 Feb 11;9:1784. doi: 10.1038/s41598-018-38346-0 (PMC6370902; doi:10.1038/s41598-018-38346-0)
Supplement: Supplementary file 1 — Supplementary Figure S1–5, Supplementary Table S1,2 [file 41598_2018_38346_MOESM1_ESM.pdf]

Empirical evaluation of variant calling accuracy using ultra-deep whole-genome sequencing data

Author list

Toshihiro Kishikawa, Yukihide Momozawa, Takeshi Ozeki, Taisei Mushiroda, Hidenori Inohara, Yoichiro Kamatani, Michiaki Kubo, and Yukinori Okada

Corresponding author: Yukinori Okada, MD, PhD (yokada@sg.med.osaka-u.ac.jp)

Supplementary Information includes

Supplementary Figures S1-5 : pages 2-6

Supplementary Table S1, 2 : page 7

Supplementary Figure S1.

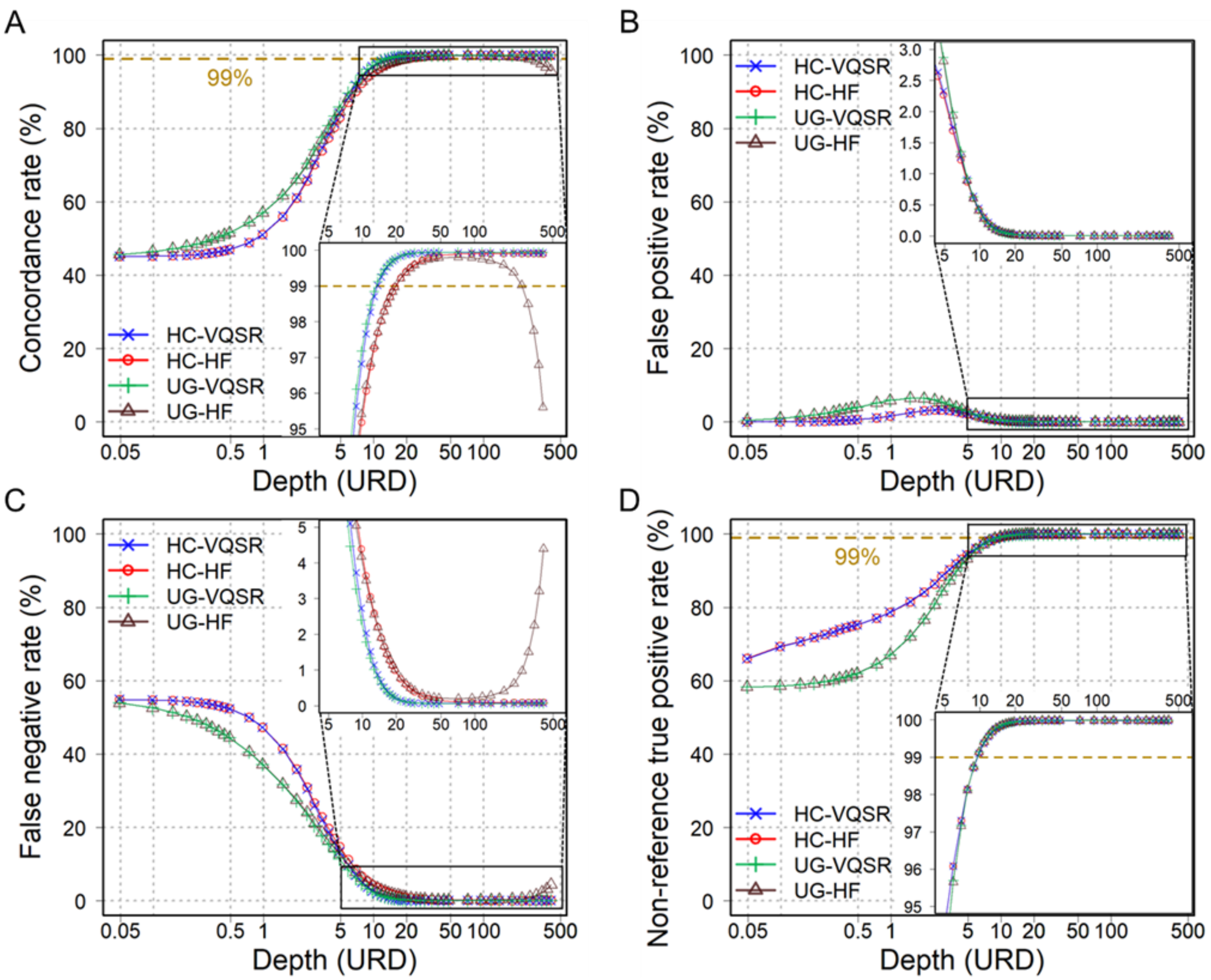

Genotype concordance comparison with SNP microarray (HC and UG data). (**A**) - (**D**) the same components as Figure 4. UG data is shown in addition to HC data. The x-axis shows the depth of each WGS data in logarithm. The y-axis corresponds to each metric of Figure 3B. (**A**) Concordance rates based on depths. In the combination of UG and HF, the concordance rates decreased at high depths. (**B**) False positive rates based on depths. In the combination of UG and HF, the false positive rates increased at high depths. (**C**) False negative rates based on depths. (**D**) Non-reference true positive rates based on depths.

Supplementary Figure S2.

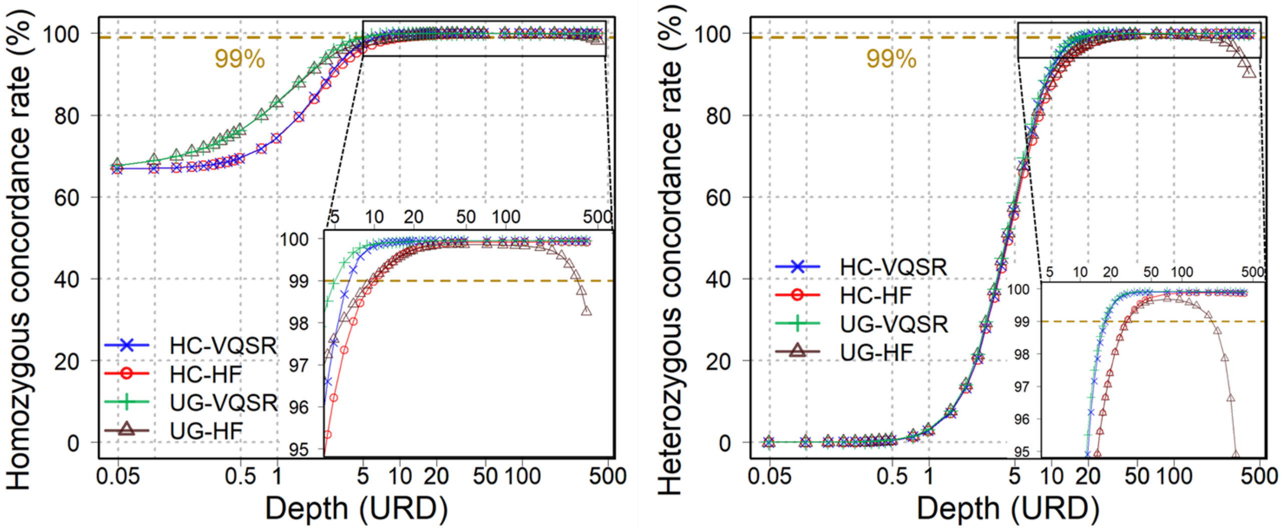

Genotype concordance comparison with SNP microarray（homozygous and heterozygous mutation). The x-axis shows the depth of each WGS data in logarithm. The y-axis corresponds to concordance rates of Figure 4A.

Supplementary Figure S3.

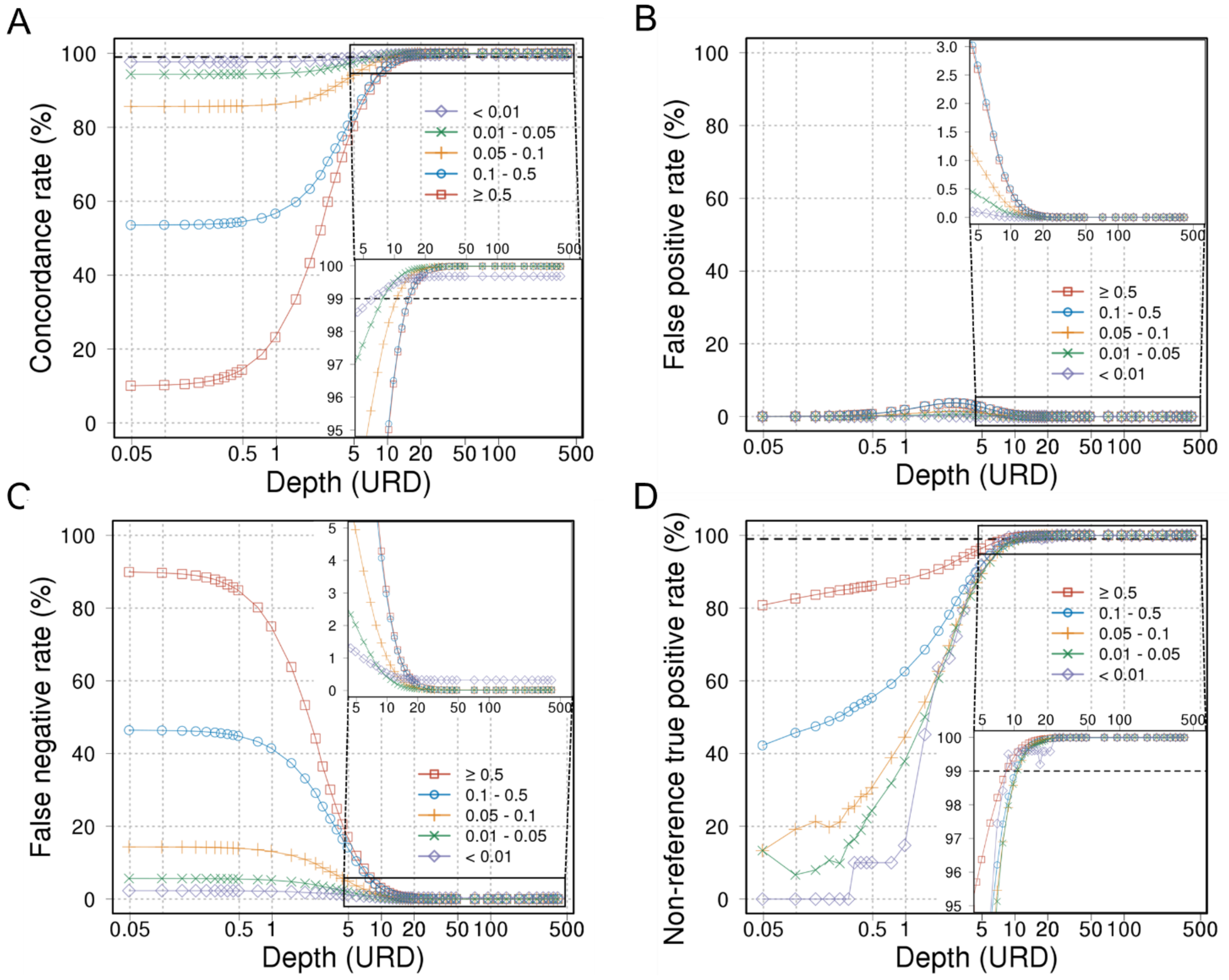

Genotype concordance comparison with SNP microarray (classification with alternate allele frequencies). The x-axis shows the depth of each WGS data in logarithm. The y-axis corresponds to Figure 3A-D. We classified SNPs into five levels of alternate allele frequencies (AAF). SNPs of low AAF showed high concordance rates at low depths, while SNPs of High AAF showed high Non-reference true positive rates at low depths.

Supplementary Figure S4.

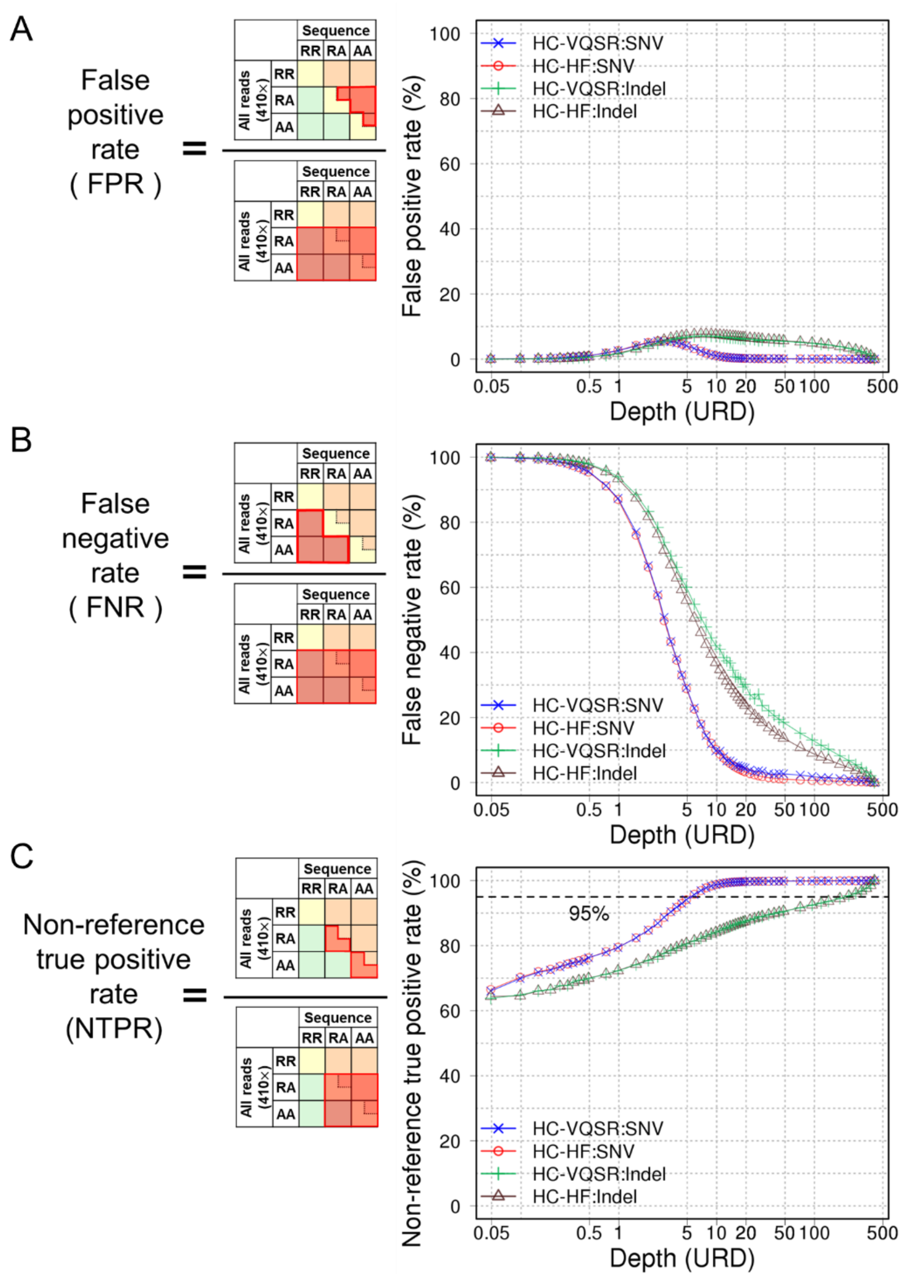

Genotype concordance comparison with WGS data of all the reads. The x-axis shows the depth of each WGS data in logarithm. False positive rates were less than 10% at any depth.

Supplementary Figure S5.

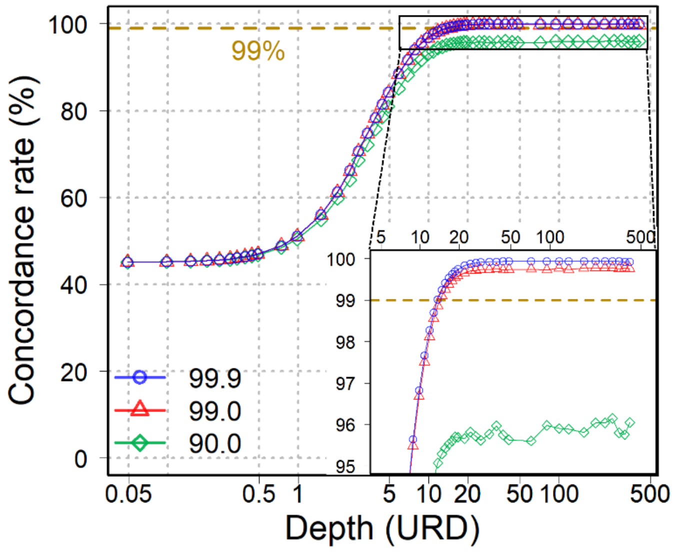

Genotype concordance comparison with SNP microarray (three levels of VQSR thresholds).

The x-axis shows the depth of each WGS data in logarithm. The y-axis corresponds to concordance rates of Figure 4A.

Supplementary Table S1.

Heterozygous and homozygous genotype concordance comparison with SNP microarray data

		HaplotypeCaller		UnifiedGenotyper	
Comparison with SNP microarray data		VQSR	Hard filter	VQSR	Hard filter
Concordance rate > 99%	All	13.7×	21.9×	13.7×	21.9×
	Heterozygous	18.6×	29.2×	18.6×	29.2×
	Homozygous	6.9×	10.8×	5.9×	9.8×
True positive rate > 99%	All	9.8×	9.8×	9.8×	9.8×
	Heterozygous	11.8×	10.8×	10.8×	10.8×
	Homozygous	0.049×	0.049×	0.049×	0.049×

Supplementary Table S2.

Genotype concordance comparison with SNP microarray data (five levels with alternate allele frequencies).

**14.7× depth data (Haplotype Caller and VQSR)**		
allele frequency	Concordance rate (%)	Non-reference true positive rate (%)
< 0.01	99.62	99.60
0.01 - 0.05	99.88	99.73
0.05 - 0.1	99.72	99.76
0.1 - 0.5	99.23	99.78
≥ 0.5	99.21	99.87
